# Health services related factors affecting the pap smear services in Fiji: a qualitative study

**DOI:** 10.1186/s12913-021-07176-8

**Published:** 2021-10-25

**Authors:** Aliti Kunatoga, Masoud Mohammadnezhad

**Affiliations:** School of Public Health and Primary Care, Fiji National University, Suva, Fiji

**Keywords:** Cervical cancer, Health care system, Screening, Pap smear, Fiji

## Abstract

**Background:**

Cervical cancer is the thirdly vast usual cause of cancer in women, and the second vast majority cause of death among women aged 14 to 44 years, both in developed and developing countries. This paper aims to explore the perception of women and Health Care Workers (HCWs) about health care related factors affecting the Pap smear services among women who are screened in the Women Wellness Centre (WWC) in Suva, Fiji.

**Methods:**

This study used a qualitative method in July–September 2019 in which women screened for cervical cancer used in–depth interviews whereas HCWs used Focus Group Discussion (FGD) in WWC in Suva, Fiji. This study used purposive maximum variation sampling where participants are selected according to the inclusion and exclusion criteria. Semi–structured open ended questionnaires were used to collect the data among participants. Data coding was done until theoretical saturation was reached. Thematic analysis was used and all the text has been coded, themes were abstracted from the coded text segments.

**Results:**

A total of 20 women screened for cervical cancer and 5 HCWs were present during the duration of the study. Health care system was a factor recognized by both groups to have been a cause for the hindrance of cervical screening. There were 8 themes identified from the study, 3 themes from the women screened for cervical cancer and 5 from HCWs. Nearly all the participants reported about the compromised of cervical cancer screening services delivery because of factors such as lack of equipment and supplies, shortage of staff, long distances to health facilities, turnaround time and delay of results which affect the uptake of cervical cancer screening services.

**Conclusion:**

While improvement has been made in the distribution of cervical cancer screening in WWC, a number of barriers and factors affect service uptake and delivery. Investments to be made in order to address the identified barriers such as turnaround time, long distances to health facilities, shortage of supplies and staff in order to improve uptake of cervical cancer screening services.

## Background

Cervical cancer is defined as an abnormal advancement of the intrauterine cells into the reproductive system of the woman [1]. Cervical cancer is a grave threat to women’s health and lives, globally where one woman dies of cervical cancer every 2 min [1, 2]. Cervical cancer is the third most cause of cancer in women, and the second major cause of death between the ages of 14 to 44 years, in developed and developing countries [3]. It accounted for 9% (529,800) of the total new cancer cases and 8% (275,100) of the total cancer deaths among females in 2008 [4].

Most of the Pacific Island Countries (PICs) have a national cervical cancer screening policy to implement cervical cancer screening programs which included a screening interval, target population, and screening methods to be used [2, 5, 6]. The screening methods routinely used include cytology, HPV testing and Visual Inspection with Acetic Acid [2]. In a mapping study by Josephine [7] it was established that few countries had data to evaluate the performance of their screening programs. From the same study it was found that cervical cancer screening coverage was generally low.

However, in a retrospective study in identifying the trends in cervical cancer using case numbers, incidence rates and case fatality in Fiji over the decade 2000–2010, found that 1234 patients were registered with cervical cancer, of whom 845 (68%) were indigenous Fijians and 357 (29%) were Indians; only 32 (3%) were of other ethnic groups. Mortality rates were much higher among Fijian women, and were far higher in women aged ≥45 years [8].

Cervical cancer mortality and incidence rates differ geographically, most cancers and associated deaths occur in less–developed region [9]. There are less studies conducted in Fiji to show the total number of women that are vulnerable of getting cervical cancer. Previous studies indicate that cervical cancer is the most recurrent cancer and most common cause of cancer deaths amongst women in Fiji [5, 8, 10–13]. Fiji has a tremendous burden of cervical cancer, with a projected age–standardized of 27.6 new cases per 100,000 women from 2003 to 2009 [10].

Studies from other regions and countries have shown the ability of cervical cancer screening to detect early signs of cervical cancer, which has contributed to improving the quality of lives of women [14–16]. Early cervical cancer detection is a very vital element when targeting to deal with the cervical cancer burden in any community. It includes the use of screening tests, normally known as “Pap smears” for identifying and treatment [17–19]. Vast majority of countries have political provision for policy modifications and adoption of programs for cervical cancer control, however a serious disconnect between operational support (implementation planning, training, budget) and national policy agendas at the local level was a regularly noted barrier to scale–up and sustainability [20]. Health care systems and quality of services provided for the patients can affect their participation in receiving health services. Knowing the factors affecting women who need cancer screening will help decision makers to organize health care systems based on patients’ priorities. Factors that can cause drawbacks to cervical screening in the health care system include governance and leadership, health financing, Health Care Workers (HCWs), medical products and technologies, health services, health information, people, communities and culture [21, 22]. A well–organized prevention service for cervical cancer considers infrastructure, trained human resources, adequate financial resources and particularizes surveillance mechanisms for investigating, screening, follow–up, and treating of the targeted women [23]. To have a better understanding of the issue, getting women and HCWs’ perception as two main target groups is essential. Their lived experience helps policy makers to adopt policies and services based on the real needs of target groups.

Not all women take advantage of the early cervical cancer screening regardless of the known benefits of it. This study aims to explore the perception of women and HCWs on the health services related factors affecting the Pap smear services among women screened for cervical cancer in the Women’s Wellness Centre (WWC) and HCWs in Suva, Fiji.

## Methods

### Study design and setting

This is a qualitative study which was carried out in WWC from the month of June to August 2019. The WWC is the central hub for areas of women’s health incorporating advocacy on family life, sexual and reproductive health, family planning, cervical cancer screening and counseling services which operates 5 days a week from 9.00 am to 4:00 pm located in the capital of Fiji, Suva. It is the largest government run reproductive health facility for the Central and Eastern division which covers the rural and urban areas and does not have a specified number of women screened because it caters for every woman who needs to be screened. All cases that are needed to be treated from other health facilities or hospitals are also referred there.

**Fig. 1:**
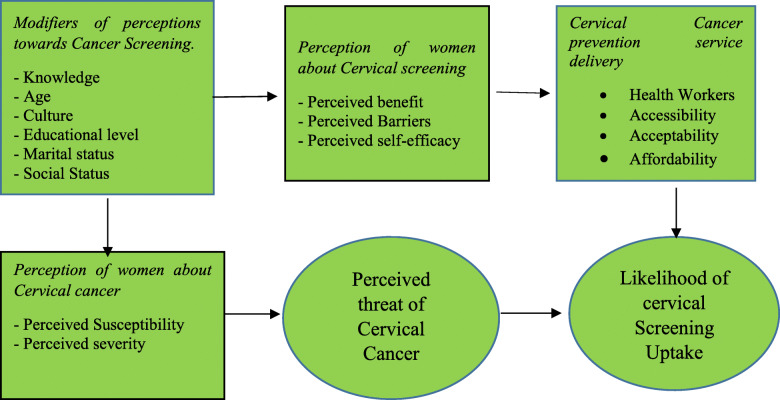
Modified and adapted from Hochbaum, 1958; Rosenstock, 1960

The concept of this research study was based and modified from the Health Belief Models (HBM) developed by (Hochbaum, 1958; Rosenstock, 1960) which predicts why people will take action to prevent, to screen for, or to control cervical cancer illness conditions. The framework is presented in Fig. 1. The framework includes factors that influence the perception of cervical cancer patients and cervical–screened patients and their likelihood of up taking the cervical cancer screening. These different factors influence each other directly or indirectly.

### Population and sample

The study population was all the patients and HCWs working in WWC Clinic. Women above the age of 18 who had at least one–time previous attendance in WWC and were screened for cervical cancer within the study period, and without mental illness were included in the study. Those who attended for the first time at WWC and were not willing to participate in this study were excluded. HCWs working in different offices at WWC with at least 6 months’ experience were included in the study. They were the doctor, nurses and midwives. Those who were not willing to participate were excluded from the study. Approximately 11 women would be attending for cervical screening per day and five health care workers would be in WWC to assist women with the cervical cancer screening.

The participants were selected using purposive maximum variation sampling where participants are selected according to the inclusion and exclusion criteria. In–depth interviews were done on 20 women who met the study criteria, and a Focus Group Discussion (FGD) was done on the five HCWs.

### Data collection tool

Semi–structured open–ended questionnaires were used to collect data from the study participants. The questionnaires were developed by researchers to address the research questions of this study. Probing questions were asked following the answers provided by the participants. There were 15 questions for women, seven questions were demographic questions and 8 questions were about the perception of women on cervical screening, factors affecting their attendance and perceived barriers. There were 14 questions for HCWs where 9 questions were on how they contribute to the prevention of cervical cancer in patients attending WWC and 5 questions were demographic questions. Before conducting the interviews, the questionnaires were given out to 3 experts for content validation.

### Study procedure

Women who met the study criteria were informed whether they wanted to participate in the study or not. They had to pick numbers from the front desk and wait until their numbers to be called out in order to be seen. They were verbally informed while waiting for their numbers to be called out in the waiting room. They were given information sheets to further inform them regarding what the study was about. Following their interest and availability, they were given the consent forms to sign and were interviewed the same day by the experienced researcher who was familiar with principles of conducting interviews. The information sheet and consent form were indifferent languages (English, Hindi and I–taukei versions) to give freedom to participants to choose any version they like. The interviews were conducted in a separate room in the WWC to ensure privacy, confidential and that all the information was attained. Each interview took 25–30 min. A bilingual translator helped the researcher to conduct the interviews in translating for those that don’t understand or speak English or I–taukei.

Five HCWs were working in the clinic but only 4 met the study criteria and were available and presented within the study period. Before the FGD was carried out, HCWs were informed to sit in a circle and the information sheets were given to them to inform them about the study. The consent forms were given out to each one of them for their signatures in participating in the study. HCWs were advised to involve the FGD in a formal manner with a clear voice since their answers would be recorded. The FGD took 60 min in a separate room during lunch hour to ensure maximum participation.

### Data management and analysis

Data collected from the digital audio recording was transcribed using hand written notes. The hand written notes were transferred to the electronic format using Microsoft word and later transcribed into Microsoft excel for data cleaning. Thematic analysis was used to dissect key words and phrases. These were then organized and consolidate per question. Each of the words and phrases was entered in one column with the participant’s code number on the column preceding it. The data was reviewed to remove any duplicate entries. Once the data was reviewed, manual coding was done to dissect text segments with the use of coding framework which will be based on the research question and the conceptual framework of the study using deductive and inductive methods to organize the data [24]. Data coding was done until theoretical saturation was reached. After all the text has been coded, themes were abstracted from the coded text segments using Attride–Stirlings thematic network analysis framework [25] and each individual cell was reviewed and assigned to a thematic area to which cell color code is applied using inductive approach.

### Ethical consideration

Ethics approval for this study was granted by the Fiji National University’s (FNU) College Health Research and Ethics Committee (CHREC) and the Fiji National Health Research Ethics and Review Committee (FNHRERC). Approvals were also obtained from the medical superintendent in charge of WWC.

## Results

### Characteristics of participants

For the women, their age ranges from 20 to 65, 10 of which are of Fijian of Indian Descent, 6 are of I–taukei and 4 are of other ethnicity. All the respondents were married. Of the 20 respondent’s majority were unemployed (*n* = 15) with a primary and secondary educational level. Most of the respondents (*n* = 16) live in the urban areas where majority of them are part of the Christian (*n* = 13) religion (Table 1).

**Table 1:** Characteristics of women (*n* = 20)

Variables	N (%)
**Age (years)**	
20–30	14 (70)
> 31	6 (30)
**Ethnicity**	
I–taukei	6 (30)
Fijian of Indian descent	10 (50)
Fijian of other descent	4 (20)
**Employment Status**	
Employed	5 (25)
Unemployed	15 (75)
**Educational level**	
No formal Education	6 (30)
Primary/Secondary	9 (45)
Tertiary/Higher	5 (25)
**Religion**	
Christian	13 (65)
Hindu	4 (20)
Muslim	3 (15)
**Area of residence**	
Urban	16 (80)
Rural	4 (20)

Majority of the HCWs were clinical nurse (50%). Their age ranges from 35 to 45, two of which are of Fijian of Indian Descent and 2 of I–taukei. Majority of them worked more than 4 years in WWC (Table 2).

**Table 2:** Characteristics of HCWs (*n* = 4)

Variables	N (%)
**Professional care**	
Medical officer	1 (25)
Public Health Nurse	1 (25)
Clinical Nurse	2 (50)
**Age (years)**	
35–36	2 (50)
40–45	2 (50)
**Ethnicity**	
I–taukei	2 (50)
Fijian of Indian descent	2 (50)
**Years of working in WWC**	
< 1 year	1 (25)
1–3 years	1 (25)
> 4 years	2 (50)

### Health system factors associated with cervical screening services

#### Women’s perceptions

Three themes were identified from analyzing women interviews including health facility distance, little space in the clinic and turnaround time.

##### Theme 1: health facility distance (navigation issues)

Locating the clinic seems to be a factor that respondents find while going for cervical screening. This was because of the relocation of WWC health care staffs to pediatric clinic due to the fire outbreak from the laundry room in Colonial War Memorial Hospital (CWMH) during the duration of the study.

*“I find it hard coming to this clinic for paps because I live far away from the city, I had to pay extra money from my pocket to come because of the transportation. I was called by a nurse to come here and check my pap smear again because they found something in my results of Pap smear.”* (45–year–old, P1)

Or another participant stated;

*“Why are they shifting clinics, every time they always change the clinic, I went to the purple building and the security told me that screening is not done there, so he showed me the way here to the MOHMS head–quarter, it takes up a lot of time just looking for the clinic.”* (32–year–old, P6)

Some participants are university students and don’t have time to look for the clinic since they have classes to attend to.

*“My friend and I came together for pap smear to the Women’s Wellness Clinic yesterday and we saw the notice on the door saying that it has been relocated so we went back home because we have no time to find the clinic, we are students in USP and we had classes yesterday so we came back after school to look for the clinic but when we arrived here, the clinic was closed that’s why we came back today.”* (22–year–old, P10)

##### Theme 2: little space in the clinic

Approximately half (*n* = 10/20) of the respondents agreed that space in the clinic where the qualitative study was carried out was a factor of why they were unable to get screened. However, they don’t see a problem of spacing in the Women Wellness Centre.

*“I feel uncomfortable coming to this clinic because back in the Women Wellness Health Centre, people don’t have to look at us or asking us questions that we don’t want to answer because only women screened for cervical cancerattend that clinic. Here we have children coming in for their check–ups and we don’t have any space to sit while waiting for our number to be called out.”* (34–year–old, P5)

Another factor is the less space in the clinic, participants have to stand outside while waiting or crown around while waiting to be screened. The screening rooms were inadequate and crowded with other services. This leads to extensive waiting time and missed chances for screening.

*“There’s little space for everyone to squeeze in”* (27–year–old, P9)

Another participant mentioned;

*“This place is so congested and we have to stand outside to wait because mothers with their babies occupy the sit.”* (33–year–old, P8)

##### Theme 3: turnaround time

Majority of the (*n* = 18/20) participants observed that Turn Around–Time was a factor why women are not being able to get screened, because all women have their own business to deal with and would have to find a time to get screened.

*“I wish we could reduce the amount of waiting hours”* (52–year–old, P13)

Participant’s admitted that the waiting hours is a factor that prevents them from getting screened because some of them don’t have all day to wait for screening.

*“Sometimes I get tired of waiting, I go back home” (49–year–old, P14)*

Or,

*“I couldn’t wait that long because my only free time from work was during lunch hour, so I need to be back to the office before the lunch hour is over.”* (37–year–old, P10)

### HCWs perceptions

There were five themes showing health system factors affecting cervical cancer screening including service delivery, flow of results, clinic infrastructure, loss to follow–up and clinic closure.

#### Theme1: service delivery

Flow of results &follow up– (Turnaround time) was of the factors the respondents believe to be the cause of why the results of cervical screening services are lagging behind.

“*From us it goes to gynae and from gynae we don’t get the results back to us, it’s either when the patient come and we check on Patis, then we know that they have been diagnosed with something or it’s from the lab. We also know from the results and from the biostatistics, we don’t know what had happened unless we check their reports on patis.”* (35 years old, female nurse).

There were not direct feedbacks from gyne clinic, only when being followed up.

*“From gynae after our cases are being referred there, sometimes only when we follow up then we can see the outcome of their biopsy results, but to get feedback from them, no, only if we ask them or follow up or even go into the Patis, then you will be able to know what is the outcome of their clinic.”* (45 years old, female nurse).

However, cytology lab sends feedbacks regarding cervical cancer positive results back to WWC.

*“We do get feedback on our positive results from the lab so that all the patients with positive results from each divisions are informed and they also inform us if some of our clients have missed their clinics so the sister in charge would call us so that we could get in touch with that patient.”* (36 years old, female doctor).

#### Theme 2: flow of results

The procedure of how the results flow was mentioned by respondents as to why the turnaround time is slow.

*“It’s usually like this, if we come across some abnormal readings, we do refer them to gynae and they do get managed there, after their proper management whatever they think is important, it can be other biopsies, they will go through procedure, after that they are followed up and then I think when they find that the patient is clear enough to have yearly or 6 months pap smears out in the health facility, that’s when the patient comes back to us and says that “gynae has finished with my procedure, this is what I had and now they are asking for us to repeat pap smear.”* (35 years old, female nurse).

Shortage of lab staffs leads to late turnaround time for results.

*“There seems to a bit delay in the turnaround time for results but understanding the concept because I spoke with the lab technician. I used to go and see her so, she took me around the lab and she explained to me at times we are the only one and specimens all over Fiji comes to us, and you know, this is the only cytology and I went in there and seen how they do it, she taken me around the cytology lab and I’ve seen that the delay is explainable and if something can be done on that. 4 weeks’ maximum is the turnaround results.”* (40 years old, female nurse).

#### Theme 3: clinic infrastructure

Unavailability of resources is one of the challenges faced in the clinic.

*“Sometimes the unavailability of resources, we sometimes face shortage of staffs when other health care staffs go for community outreach. Sometimes only one staff will stay behind and look after women who come in for cervical screening and it takes up a lot of time” (35 years old, female nurse).*

It was also pointed out the shortage of equipment’s and consumables.

*“Some of the challenges that I face is the shortage of equipment’s and consumables that are needed for pap smear.” (36 years old, female doctor)*.

Difficulties in accessibility to pharmacy or medicine were also highlighted.

*“For us, in the wellness center for women..for treatment, we need to give patients our prescription to get it from the pharmacy and sometimes the drugs are out of stock, the ones that we need for our clients to be treated with, but otherwise they have to buy it from the chemist in town and another thing is, right now we are out of stock with thin prep since the second week of May until today, so we are lucky to be provided some from this US team who are here in the clinic, if not there will be no pap smear done, we just start our pap smear again early this month July.”* (40 years old, female nurse).

#### Theme 4: ignorance to feed–back results

Loss to follow up with screened women is another factor faced by health care staffs in their work area.

*“Another thing that we are facing is the patients especially for follow up and the address are not fully specified as you know, there are students who also visit our clinic so when we explain to them to be specific with their address so we can easily notify them, they usually take it lightly without telling us their specific address or even when we told them to come for counseling, they will always say that they will turn up but they would never come.”* (36 years old, female doctor).

The belief that having positive result is death warrant if they are tested positive so it is better not to go for screening.

*“Some women think that their results will come out positive, so they will tell us they would come to get their results, and come for their next appointment but they never appear. Sometime when we contact them, they won’t answer their phone which makes it difficult for us to let them know about their results”*

#### Theme 5: clinic closure

Closure of the clinic due to maintenance was a challenge for the respondents.

*“However, the working environment is okay but right now our clinic is closed due to some maintenance being done, that’s why we were being shifted to this clinic and share the facility with the Suva MCH clinic and we are lucky that they are giving us the space.”* (45 years old, female nurse)

Clinic to be used as a storage room.

*“We were told that after the maintenance of the clinic, it will be used as a storage room for the Colonial War Memorial Hospital, so we have to work with any clinic that has been told for us to work in”*

Shifting from one clinic to another was also a factor pointed out by the health care staffs that result in less women coming in for screening.

*“We have been moving from facility to facility and this leads to less women coming in for screening. Most women know the location of the WWC but when it was closed, many women never turned up for screening.”*

## Discussion

This study provides information about health system factors affecting cervical cancer screening. Moreover, the study was carried out among women in selected health facility in Suva, Fiji and provides qualitative information on the perception of the health system factors affecting cervical screening among women and health care staffs.

In this study health facility distance was one of the factors screened women in WWC discussed to have been the reason why they are not attending screening. Women in the rural areas consider the distance from where they are staying to the location of the clinic to be very far [26, 27]. Most women had to travel for hours in order to get to the clinic, and with distance they have to pay extra from their pocket in order to reach their destination. In line with the results of this study, a study showed that prevention and knowledge of cervical cancer (95%CI = 2.14–16.03, OR = 8.90) and distance to the facility building which offers cervical cancer screening (95%CI = 0.18–5.10, OR = 3.98) were also found to be significantly associated with screening uptake [28].

This study also found that transportation does not travel to town daily and with that, they had to wait because the transport runs on schedule or else they have to hire a transport in order to reach their destination. Moreover, among women living in underprivileged areas, those that lived nearer to clinics probably doubtless to have a mammogram was found in a study conducted in Australia [29]. Distance and travel time to screening building facilities was also shown to be reciprocally related to neighborhood poverty line [29]. Nevertheless, other studies showed that there is a weak positive association in a county with minimal use of public transport in the U.S. [30].

Moreover, women screened for cervical cancer also mentioned the little space and the turnaround time for health care staffs in the clinic to be a factor that influence them not to go for cervical cancer screening that is in line with other studies’ findings [23, 31]. A study conducted in Kenya the barriers to screening were examined through a survey of 106 healthcare staff and the frequently cited barriers was the insufficient space and waiting time [32]. Women’s domestic roles, including their responsibility for the household chores and caring for the family, meant that they were unable to attend clinics for cervical cancer screening and thereby risked neglecting their health. Most health care government run facilities face these factors. Women are free and are entitled to choose the health care facility they want to get screened in, however WWC is mostly attended because of the great services they provide to women.

Adding on, another factor that affects the cervical screening services is the delay in results which are not sent directly to the clinic, only when the clinic needs the results to be given to the patients, or results being checked directly on Patis. The most frequent technique used for diagnosis and early screening of cervical cancer is pap smear. Nevertheless, there are many reasons to the delaying of results, where first of; inaccuracy in the manual analysis of the pap–smears is likely to due to human mistake and time–consuming, the process is also tiresome [33]. Fiji is a developing country and is yet to upgrade on technology [34]. Hence, other countries are developing tools for auto– mated diagnosis and categorizing of cervical cancer from pap–smear images [35], suggesting approaches for automatic identification of cervical cancer cells in images that are taken from thin liquid based cytology slides [36] and using advanced techniques which mainly contributes to work– flow and reduces time [37].

Furthermore, flow of results was also a factor cervical cancer screening results are delayed in this study which might be due to the routinely screen samples daily by cytologist to be clarified positive or abnormal at a certain percentage [18, 38, 39]. Also the shortage of lab staffs is another factor that are preventing health care staffs provide cervical screening services to women screened for cervical cancer as highlighted by other similar studies [31, 32]. Sometimes there are usually a few health lab staffs in the lab which lags behind cervical screening results. This study is similar to a study conducted at Uganda with the aim of evaluating the factors that are associated with the uptake of cervical screening among HIV infected women at Mildmay Uganda which also shows the shortage of staffs as a barrier to screening [40].

In addition, shortage of equipment’s, was also a factor the health care staff’s respondents believe to be the cause of why the results of cervical screening services are lagging behind and the lack of resources in the facility such as shortage of equipment’s and drugs. This study was related to the study conducted in Peru with the aim of identifying multiple barriers to obtaining cervical cancer screening, follow–up, and treatment which showed two major problems that prevailed across the health system; the widespread lack of resources and the centralization of cervical cancer treatment, it also found that patients were not receiving proper treatment because neither the referral system nor the treatment procedures were systematic [41]. Similarly, with the study conducted in Kgatleng district, Botswana [42] and sub–Saharan Africa [43], where health system factors point up the inconsistent appointment systems, unavailability of results, equipment shortages and long queues. Additionally, the findings of this research are not different to other studies on lack of access to cervical cancer screening [5, 8, 12–16, 20, 21, 44].

Loss to follow up on women going for screening was also a factor health care staff’s face while trying to explain to women the importance of their results of screening. Women tend to take screening lightly and some are never specific with their home address or contacts. This might be due to past experience from relatives centered on fear of results or embarrassment [45, 46]. However, turn around–time was also a factor mentioned in which many technicians would wait to analyze samples until they had accumulated enough to warrant an overtime payment. This was further supported by a study with the aim of looking at the position of HPV vaccine in the prevention of cancer of the cervix showed that combining pap smear screening methods and HPV vaccine specifically to high risk group would greatly decrease the mortality and morbidity connected with cancer of the cervix [47]. In addition, Slow turnaround time of results, mismanagement of information, such as loss of results and poor quality smears rejected by laboratories could be a reason resulting in the late feedback of results from technicians [37].

However, health care staffs in the clinic also were being shifted from time to time to other nearby clinics because their clinic is being used by other health care staffs from other fields. Women screened are frustrated in the shifting of clinics because it also took up their time in locating other clinics where staffs are being shifted to. In the duration of this study, the WWC was being shut down due to maintenance of the clinic and the health care staffs working in the WWC were shifted to the child health clinic which was the closest clinic to WWC. Screened women find it hard in locating the clinic which takes up much of their time waiting for hours [32], which was also a reason as to why they don’t get screened. Moreover, a study conducted with the aim of quantifying the opportunity costs related with cervical cancer screening in young women attending planned parenthood clinics found that time is associated with cervical cancer screening which represents an important opportunity cost and should be considered in studies attempting to identify barriers to screening adherence [15]. However, one of the reasons in closing the clinic is to be used as a storage room for the main hospital. On the other hand, shortage of buildings or clinics for the use of cervical cancer screening can be overcome by introducing cervical cancer screening to all health clinics so that women can access cervical cancer screening in every clinic. It conjointly avoids overcrowding and although many studies have used hospital and clinical models to evaluate, describe and define, health–navigator programs [48, 49], only a few studies have observed community–based settings health–navigating programs [49, 50]. These community conditions are important because civil society organizations promote various cancer screening and education programs aimed at overcoming many environmental barriers and restrictions [50].

### Limitations

Only one FGD was used to collect data among HCWs. This might affect the data saturation among HCWs. Also this study was carried out in an urban setting, so more women leaving in the city are mostly screened compared to the rural areas. Selection of women screened for cervical cancer and health care workers in rural settings and in remote areas might shed more light on the factors related with cervical cancer screening.

## Conclusion

Significant progress has been made in the early treatment services and delivery of cervical cancer screening. The total number of health buildings offering cervical cancer screening services has increased since the opening of WWC, alongside health care staffs that have been trained to provide screening services. The value of services being delivered somehow has been compromised by the lack of supervision of managers at the district level which leads to the shortage of human resources, lack of adequate supplies and appropriate equipment to successfully provide cervical cancer screening services. Investments is required to be taken care off in order to address these barriers. Eliminating these barriers is paramount if we want to achieve the goal of reducing the mortality and incidence rates of cervical cancer. Therefore, it is vital to bring forth cervical cancer screening services in all health clinics, to enable women and involve women in cervical cancer screening.

## Data Availability

The data that supports the findings of this study are available on request from the corresponding author.

## Abbreviations

CHREC: College Health Research and Ethics Committee
CWMH: Colonial War Memorial Hospital
FNHRERC: Fiji National Health Research Ethics and Review Committee
FNU: Fiji National University
FGD: Focus Group Discussion
HCWs: Health Care Workers
HPV: Human Papillomavirus
WWC: Women Wellness Centre
